# A Physiological Study of Tobacco and Its Action upon the Organism

**Published:** 1904-01

**Authors:** Georges Petit


ATLANTA

### Journal-Record of Medicine.

Successor to Atlanta Medical and Surgical Journal, Established 1855, and Southern Medical Record. Established 1870.

VOL. V. JANUARY, 1904. No. 10.

BERNARD WOLFF, M.D., EDITOR, Nos. 319-20 Prudential.

Published Monthly.

M. B. HUTCHINS, M.D., BUSINESS MANAGER, 1007-1008 Century Bldg.

### ORIGINAL COMMUNICATIONS.

### A PHYSIOLOGICAL STUDY OF TOBACCO AND ITS ACTION UPON THE ORGANISM.

BY DR GEORGES PETIT.

(Translated from *Le Progrès Médical,* by Bernard Wolff, M.D.)

Tobacco owes its toxicity to several substances of which the best known is nicotine. Nicotine is an alkaloid, liquid, colorless, soluble in water, of acrid taste. It exists in all parts of the plant (Vauquelin 1809.) The quantity of nicotine in tobacco varies a great deal; its percentage ranges from 2 to 8, depending upon the individual plant and the country in which it is cultivated. It is an active poison, one of the most violently toxic of the known alkaloids; prussic acid and aconitine alone are comparable to it in rapidity of action. If two drops of nicotine be placed on a dog's tongue, the animal is seen to make efforts at swallowing, almost immediately falls in violent convulsions and dies in less than one minute. The fatal dose of nicotine is between 20 and 21 milligrammes for each kilogramme of the animal's weight. In man one drop of nicotine produces grave symptoms. Eight drops are sufficient to kill a horse. "In whatever way nicotine is given, by the mouth, applied to an abraded surface, introduced under the skin, or instilled in the conjunctiva, the animal is at once overcome. He dies in exceedingly violent convulsions. Horses are put in a frightful condition; they stand with limbs extended, become furious and uncontrolable, fall and lie down making convulsive and disordered movements." (Claude Bernard.)

A part of the nicotine occurs in the natural state in tobacco smoke, another part is decomposed by the combustion of the tobacco. But there are several products resulting from this combustion. The substance may be modified, not destroyed, by burning. The physiologist, Le Bon, has been able to isolate, administer and experiment with, a series of five new products from the combustion of nicotine, and singular as it may appear the substances were all much more powerfully toxic than nicotine itself. Besides nicotine there are found in tobacco smoke ammonia salts, prussic acid, oxide of carbon, collidine, nicotianine, substances on the base of pyridine, as well as organic bodies, resins and acids. The puffs which smokers take from their cigar or pipe contain sufficient nicotine to kill them if it were condensed and absorbed entire. That it does not at once kill them will be seen from the following reasons: Nicotine reaches the mouth distilled but in a state of warm vapor. It rarifies and mingles to a considerable extent with the warm air which forms the smoke. Almost imperceptible molecules of it impinge upon the buccal mucous membrane which is warm; it therefore does not condense sufficiently to be absorbed; for this reason its harmful action is slow rather than rapid. It would be otherwise if the mouth were cold. The nicotine would be condensed and though the smoke remained but a short time in the mouth the smoker would be overcome as is the case in cold-blooded animals, snakes, lizards and frogs which are instantly killed by a puff of smoke blown in their mouths. It is this property of nicotine of condensing upon cold surfaces that renders some mouthfuls of smoke blown in a glass sufficient to poison and bring near to death a person who takes from it. (Depierris.)

Nicotine occupies a place between the vaso-motor poisons like potassium bromide and cardiac poisons like digitalis. It is classed with belladonna among the poisons which affect both the heart and the vessels, cardio-vascular. (Blatin.)

Numerous observations on animals have been made. Orfila was the first observer in 1843, then Claude Bernard, Vulpian, Wrothnagel, Rossbach, Rosenthal, and others. Observations on the effects of nicotine on man have been made by the clinicians Germain Sée, Depierris, Blatin, Gruby, Dujardin-Beaumetz, Magnan, Merlin and others. Clinical observation and experimentation have established the fact that the most varied organic and functional disturbances are recognized among individuals suffering from chronic nicotine poisoning.

The first conclusion which may be formulated from our present knowledge is this: that like other poisons tobacco prepares the soil for the evolution of disease. This axiom is to-day well established and may be taken as a dogma—one which only blindness or absolute ignorance would fail to acknowledge. Tobacco smoke contains a portion of nicotine and according to the researches of Dr. Lefèvre, Professor in the University of Louvain, the smoker absorbs a part of this nicotinized vapor. The fact is indubitable that absorption may occur in two ways. The first of which is by the digestive tract. It is probable that a part of the nicotine in the gaseous state remains in the mouth and is carried in the stomach in the act of deglutition. But it is especially through the pulmonary tracts that absorption occurs. It is well known with what facility gases are absorbed from the lung surface. This occurs when chloroform passes in the blood for the purpose of anesthetizing a patient. A certain amount of chloroform must rapidly pass into the blood to attain this purpose. The same result can be obtained in like manner by absorption through the digestive mucosa, but the absorption is much slower than through the air passages. It may be remarked that it is not the smoker alone who absorb the nicotine. When smoking is going on in a more or less confined place, one absorbs the poison not only on his own account but it is distributed among other persons present who, while not themselves smoking, respire an atmosphere charged with the fumes of tobacco.

Among the well known risks incurred by the abuse of tobacco is that of angina pectoris. It occurs with chronic nicotine poison. Tobacco can directly cause a spasm of the coronary arteries as well as an excitation of the nerve plexus. One may probably recall instances of this kind occurring in those who, without smoking, remain for some hours in a small space where many are smoking. In an atmosphere of tobacco smoke one is obliged to inhale the noxious products of the fumes which even penetrate the alveoli of the lungs and are absorbed in larger quantities than when the smoke is simply drawn into the mouth and blown out again. The condition of chronic intoxication creates a sort of latent tabagism. On this point numerous controversies have arisen among different observers. It is a subject the understanding of which does not appear to be easier than the foregoing. Certain people tolerate very large doses of the poison for a long time. It is impossible to admit a toxic quotient any more than a therapeutic quotient in a mathematical proportion. The variations are as numerous as individualities and one is unable to give the measure of the quantity consistent with the health of the individual. All figures given in this connection must be regarded as fanciful.

Each one of us has organs more or less resistent, or more or less weak (LeBon). Given a poisonous substance which is capable of producing effects, the effects fall first upon the least resistent, or in other words it acts equally upon all, but the least resistent suffer first. Very diverse elements come into play, individual resistence, idiosyncrasy, special susceptibility, and the like. This is well brought out by Peter in the chapter on Angina Pectoris in his work on diseases of the heart: "Undoubtedly all smokers do not suffer angina pectoris, but it has been demonstrated that it is prone to occur in old, inveterate smokers, that is to say, in those who possess at the same time an old aorta, an old heart, and old cardiac nerves." This bears out, with the researches of pathological anatomy, the truth of Cazalis' classical phrase, "A man is as old as his arteries." According to Huchard the immoderate use of tobacco produces a spasm of the coronaries, ending in three varieties of angina pectoris: sclero-tabagic, spasmo-tabagic and gastro-tabagic.

In general smokers support the abuse of tobacco for a more or less long time without experiencing its ill effects, but some fine day symptoms of intoxication make themselves felt when the subject diminishes the daily amount consumed. The toxic effect is due to an accumulation of poison in the system and a diminished resistance on account of age. The heart and the nervous system, being the organs most susceptible to the poison, are the first to manifest the appearance of trouble. In these conditions it is easy to understand that in individuals previously free from any pathological lesion the first morbid manifestation of toxic origin can serve as the point of departure of an indelible pathological affection. It is in this manner that tabagism can put arterio-sclerosis on the way to development. It would appear that tobacco has a more marked action in the nervous and arthritic.

From use to abuse there is but one step, and the lack of definite limits between the two very different conditions makes it a matter of experience and discernment of those concerned. The abuse of tobacco exercises a terrible effect upon the health and intellectual faculties. When the craving for smoking has become established, it is very difficult for the smoker, even though he realizes the effects, to resist, because the intelligence is dulled, memory lessened, perception less rapid, the mind has lost its energy and the victim is not free to direct his brain. He has lost the function of directing, and this condition reduces the mentality to a state of decrepitude—*aboulia*. Under the sway of his obsession the smoker, while realizing fully the danger that he runs, has not the force of energy sufficient to cast from him the poison which is killing him and which he seems to lack the power to resist.

The same phenomena occur with opium, morphine, cocaine, alcohol, and all such toxic substances, and it is the first indication of poisoning of the nervous system. It is an incontestable fact that nicotine intoxication is very persistent and that those who are poisoned by tobacco remain for a long time under the influence of the poison. It is easy to perceive this when one considers the facility with which the symptoms reappear in former smokers on the least excess.

When tobacco is administered experimentally to animals it produces serious morbid changes, alterations in the hepatic cells, diminution in the size of the liver, sclerosis, and absolute diminution in weight. In the blood it produces a decided reduction in hemalin. The nervous system undergoes changes. There is a peripheral neuritis with congestion of the neuroglia. Nicotine especially affects the cardiac nerves. Tabagism produces of itself a characteristic form of angina pectoris and aggravates the symptoms of angina with lesions. Along with other nervous effects of tobacco are to be included circulatory disturbances with or without alteration of the blood. The motor ganglia of the heart, those of Bidder, Ludwig and Remack, are affected by the action of the poison on the nerve tissue, and in this way the effect falls indirectly upon the heart, the circulation and the blood.

Further, nicotine is a poison which hinders hematopoesis and it retards the absorption of oxygen by the hemaglobin.

The process is analogous to that which occurs in oxycarbon intoxication when the oxyhemaglobin is replaced by carbo-oxyhemaglobin.

Tobacco reduces oxyhemaglobin by its carbonic acid (this can be shown by the spectroscope), and in giving back its oxygen even by mingling in the air, oxyhemaglobin reforms. When this action is prolonged oxygen is driven off and the reduced hemaglobin decomposed. The volatile fatty acids contained in tobacco, the tartaric, citric, prussic acids decompose hemaglobin in giving rise to coloring matter which contains no trace of iron, as the iron becomes separated and precipitated as an oxide.

Under the influence of nicotine the blood-globule loses its vivifying power, and this phenomenon is observed in old smokers by their leanness, yellowish complexion, cachexia and the like. It is seen especially among the poor and alcoholic in whom the tendency to thinness is not overcome by abundant nutrition. It also has the effect of lowering the temperature. The lung is directly affected by the action of the fumes of tobacco. The penetration of the toxic principle takes place by two routes, through the ramifications of the bronchial tubes and through the capillary blood-vessels. Nicotine in a small dose quickens respiration by stimulating the cord, bulb and pneumo-gastric. In large dosage, on the contrary, it lessens respiratory movements by a paralysis of the vagus.

In a former article I have shown that nicotine in regard to tuberculosis has no bactericidal power and does not hinder the multiplication of the tubercle bacilli. Tobacco exerts a direct and marked influence upon the development of pulmonary tuberculosis. It weakens the organs and alters their functions and diminishes their power of resistance. In this way a condition of morbid receptivity is created which prepares the system for the invasion and reproduction of the tubercle bacillus. The action of tobacco upon the digestive tract, and especially upon the stomach, was long ago established by experimentation and confirmed by clinical observation. It is very evident that tobacco which has such a profound influence upon the pulmonary and cardiac branches of the pneumogastric would be hardly likely to spare the gastric branches of this nerve.

According to the experiences of Blatin, the effect of tobacco upon the vagus is to produce at first an excitation which is followed by a paralysis—in other words, the same phenomena as are observed when the nerve is first stimulated, then cut.

Has the mode of smoking any influence upon its action?

It is generally considered that cigarettes are the most harmful on account of the paper. This is an error which I feel should be corrected as briefly as possible. The paper contains only an insignificant and entirely harmless amount of cellulose, and though Dr. William Murrell once showed the presence of arsenic in it, this is exceptional, probably unique. The capital consideration is the effect of the tobacco itself, and whatever be the mode of using it, by pipe, cigar or cigarette, the intoxication always manifests itself in the same *modus agendi.* While cigarette smoke is usually inhaled by the smoker—this is merely a secondary detail. It may be readily affirmed that the ill effects are similar among all varieties of smokers. The constitutional effects fall upon the nervous system and consecutively upon the blood. Very manifest symptoms of a general intoxication are observed when the local symptoms are absent or but slightly marked.

Tobacco is a general poison, the effects of which are felt throughout the whole economy through the intermediation of the nervous system. It acts in the same manner as opium and alcohol without leaving any traces at its point of introduction.

